# Metabolic syndrome and chronic kidney disease

**DOI:** 10.4103/0971-4065.41279

**Published:** 2008-01

**Authors:** D. Bhowmik, S. C. Tiwari

**Affiliations:** Department of Nephrology, All India Institute of Medical Sciences, New Delhi, India

**Keywords:** Chronic kidney disease, metabolic syndrome, diabetes mellitus, obesity

## Abstract

Obesity is fast becoming a bane for the present civilization, as a result of sedentary lifestyle, atherogenic diet, and a susceptible thrifty genotype. The concept of metabolic syndrome, which is a constellation of metabolic disturbances, has crystallized over the last 80 years with the aim of identifying those at greater risk of developing type 2 diabetes and cardiovascular disease. These patients have visceral obesity and insulin resistance characterized by hypertyriglyceridemia. Recently, it has been realized that they are also at an increased risk of chronic renal disease. Release of adipocytokines leads to endothelial dysfunction. There is also activation of systemic and local renin-angiotensin-aldosterone system, oxidative stress, and impaired fibrinolysis. This leads to glomerular hyperfiltration, proteinuria, focal segmental glomerulosclerosis (FSGS), and ultimately end-stage renal disease (ESRD). Treatment consists of lifestyle modifications along with optimal control of blood pressure, blood sugar and lipids. Metformin and thiazolidenidiones reduce insulin resistance; while angiotensin converting enzyme inhibitors and angiotensin receptor blockers reduce proteinuria and have a renoprotective effect. Exciting new medical therapies on the horizon include rimonabant a cannabinoid receptor type 1 antagonist, soy proteins, and peroxisome proliferator-activated receptor (PPAR) agonist. Bariatric surgery for morbid obesity has also been shown to be effective in treating metabolic syndrome.

## Introduction

Obesity is a global problem. In the USA two-thirds of the population is overweight or obese. In the urban areas of India too, obesity is rapidly increasing. The prevalence in children has also been rising at an alarming rate. Genetic predisposition (the so-called Thrifty gene hypothesis) coupled with a sedentary lifestyle and atherogenic diet is probably driving this pandemic. Obesity produces several adverse health consequences including diabetes, cardiovascular disease, stroke, osteoarthritis, sleep apnoea, and reduced life expectancy.^1^ Over the last few decades, the aim has been to correctly identify and intervene in those individuals who are at an increased risk of diabetes and cerebrovascular disease. Clustering of metabolic disturbances, hypertension, hyperglycemia, and gout, was put forth in 1920 for the first time.^2^ More than two decades later, upper body adiposity was added. Reaven in 1988 used the term Syndrome X.^3^ He stated that insulin resistance, a concept first introduced half a century earlier, plays a vital role in determining who will and who will not develop coronary artery disease. The following year Kaplan used the term Deadly Quartet for the combination of upper body adiposity, glucose intolerance, increased triglycerides, and hypertension.^4^

## Definitions of Metabolic Syndrome

Currently, the widely accepted term is metabolic syndrome. It has been seen that the prevalence of metabolic syndrome increased with the severity of obesity and reached 50% in severely obese individuals.^2^ Over the last decade it has been defined differently by World Health Organization (WHO), National Adult Education Programme Adult Treatment Panel III (NCEP-ATP III), and the International Diabetes Federation (IDF) [Table 1].^5^ Although the central theme remains the same, there are some vital differences amongst the three diagnostic criteria. While according to the WHO criteria insulin resistance is a must for diagnosis of metabolic syndrome, upper body adiposity is requisite for satisfying the IDF criteria. It is pertinent to note that microalbuminuria has been accepted as one of the WHO criteria. While the estimated prevalence of metabolic syndrome estimated by the different criteria is similar, often the individuals identified are different. Most of the epidemiological studies have used the NCEP-ATP III criteria as they are simple to use in the clinical setting. The prevalence of metabolic syndrome varies widely according to the geographical location, race, gender, and urbanization, ranging from a low of 8% in French males to a high of 60% in the female Native Americans. In India, the prevalence is 15-20%.^2^ South Asians are at a greater risk for developing complications as compared to Americans. Hence, the cut-offs for diagnosis of metabolic syndrome as per the IDF definition have been kept lower.

**Table 1:** Criteria for diagnosis of metabolic syndrome by three commonly used definitions

Criteria	NCEP-ATP III	WHO	IDF
	At least three or more of the following	Glucose intolerance, IGT or insulin resistance plus two or more of the following	Central obesity* plus two or more of the following
Fasting BSL	>100 mg%	-	>100 mg%
BP	>130/85	>140/90	>130/85
Triglycerides	>150 mg%	>150 mg%	>150 mg%
HDL chol	Males < 40 mg%	Males < 35 mg%	Males < 40 mg%
	Females < 50 mg%	Females < 39 mg%	Females < 50 mg%
Obesity	Males > 102 cm		
	Females > 88 cm	W/H ratio males > 0.9, females > 0.85, and or BMI > 30	As above
μ-Albuminuria	-	UAER > 20 μg/min or Ualb-creat ratio ≥ 30 mg/g	-

* Central obesity is ethnicity specific, *USA*: as per NCEP-ATP III males ≥ 102 cm, females ≥ 88 cm, *Europoids*: males ≥ 94 cm, females ≥ 80 cm, *South Asians and Chinese*: males ≥ 90 cm, females ≥ 80 cm, IGT: Impaired glucose tolerance, W/H: waist/hip, UAER: Urinary albumin excretion rate

## Pathophysiology of Metabolic Syndrome

### Central obesity and dyslipidemia

As mentioned earlier, combination of genetic and environmental factors leads to central obesity. This hyperplastic and hypertrophic mass of adipocytes plays multifactorial and vital role in the pathophysiology of metabolic syndrome. There is increased flux of free fatty acids into the liver leading to excessive hepatic production of triglycerides and resultant hypertriglyceridemia. Also, adipocytes secrete inflammatory cytokines like TNF-α, IL-6, and C rective protein (CRP resulting in endothelial dysfunction; while there is relative deficiency of the anti-inflammatory cytokine adiponectin.^6^

### Insulin resistance and glucose intolerance

The contribution of insulin resistance to the pathogenesis of metabolic syndrome was first elucidated by Reaven.^3^ Insulin resistance is characterized by fasting hyperinsulinemia to maintain euglycemia. There is also an inability to suppress glucose production by the liver and the kidney; and also to mediate glucose uptake by the muscle and adipose tissue.^2^ Hypertriglyceridemia is an excellent marker of insulin resistance. Besides, hyperinsulinemia leads to increased sodium and uric acid reabsorption by the tubules leading to hypertension and hyperuricemia. Ultimately there is β-cell burnout and development of diabetes.

### Leptin deficiency

Leptin deficiency or resistance is associated with triglyceride accumulation in the liver and muscles due to inability of leptin to activate adenosine monophosphate (AMP) kinase in muscle.

## Mechanism of Renal Disease in Metabolic Syndrome

Insulin resistance and the release of inflammatory cytokines lead to glomerular mesangial expansion, basement membrane thickening, podocytopathy, and loss of slit pore diaphragm integrity. The other contributory factors include endothelial dysfunction, renin-angiotensin-aldosterone-system activation, oxidative stress, and elevated plasminogen-activator-inhibitor-1. Ultimately, the end result is glomerulosclerosis and tubulo-interstitial injury.^6^

## Renal Abnormalities in Metabolic Syndrome

Studies in obese Zucker rats have elegantly demonstrated the pathology of renal disease in metabolic syndrome.^7^ These rats have a defect in the brain receptor leading to hyperphagia, obesity, hypertension, insulin resistance, and dyslipidemia; thus closely mimicking metabolic syndrome in humans. They have hyperfiltration and ultimately develop glomerulomegaly and FSGS.

Studies in humans have confirmed these findings. Chagnac *et al*.^8^ demonstrated that in patients with severe obesity, GFR was increased by 50% and renal plasma flow (RPF) by 30% as compared to lean controls. Conversely 17 morbidly obese patients with body mass index (BMI) >48 who lost 48 kg in 1 year after bariatric surgery, GFR and RPF decreased although their BMI was still 32 kg/m^2^.^9^ In a recent study^10^ in young healthy males with a mean age of 18 years, metabolic syndrome was found to be associated with 6.9-fold increase in odds ratio (OR) of glomerular hyperfiltration. Also these patients had high-leptin levels. They concluded that glomerular hyperfiltration has an early-onset in life, much before manifestations of cardiovascular disease; and so may be a marker of metabolic risk. It is well known that hyperfiltration (even in nondiabetic patients) leads to proteinuria. Chen *et al*.^11^ has shown that there is a graded prevalence of microalbuminuria according to the number of metabolic syndrome components. Similar findings have been reported from Japan.^12^ Kidney biopsy studies in morbidly obese subjects have demonstrated FSGS.^13^ The important feature differentiating FSGS in obesity from idiopathic FSGS was the universal presence of glomerulomegaly (226 vs. 168 *μ*m). However, in obese patients foot process effacement was less and the long-term outcome better.

**Fig. 1:**
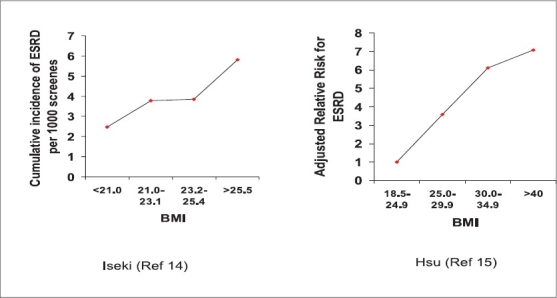
Body mass index and the risk of ESRD

**Fig. 2:**
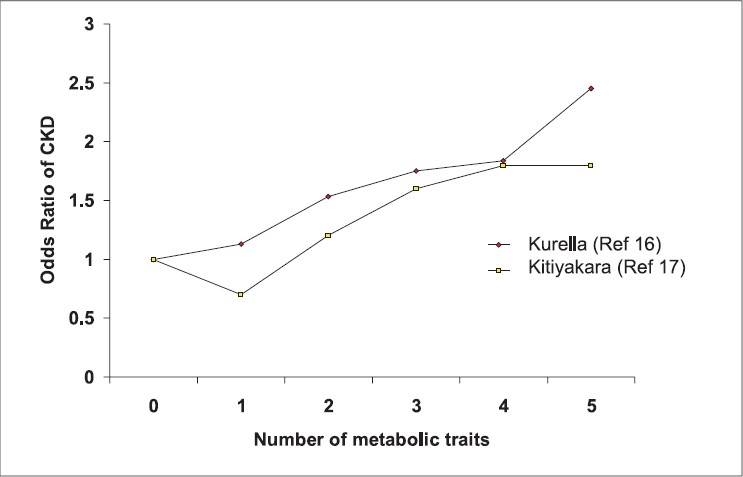
Correlation of number of metabolic traits and risk of chronic kidney disease

Studies^14^,^15^ have shown that higher the BMI more the prevalence of ESRD, after adjustment of BP and presence of diabetes. Iseki *et al*.^14^ studied the development of ESRD in about 100,000 subjects after 17 years and found that there was a graded increase in the number of screenes who developed ESRD [Fig. 1]. In another large study Hsu *et al*.^15^ followed about 300,000 subjects and found that the relative risk of developing ESRD increased substantially as the BMI increased [Fig. 1]. The authors concluded that high BMI was a strong and potentially modifiable risk factor for ESRD. Both these studies had been initiated more than two decades ago and did not study the other components of metabolic syndrome. In a recent study^16^ of 10,000 nondiabetic USA subjects with a normal baseline GFR who were followed up for 9 years, the adjusted risk of developing chronic kidney disease (CKD) was 43% higher in participants with the metabolic syndrome. Similar findings have also been reported from South-east Asia.^17^ Metabolic syndrome was associated with increased risk of CKD at baseline and also of developing new CKD after 12 years follow-up. Moreover both the studies showed that there was a significant graded relationship between the number of metabolic syndrome components and risk of CKD [Fig. 2]. A cross-sectional survey in the Chinese population has also concluded that metabolic syndrome might be an important risk factor for CKD.^18^

The main utility of the concept metabolic syndrome lies in the fact that it is a very powerful public awareness tool. Almost 80% of obese children become obese adults and hence are exposed to the metabolic consequences of obesity for a very prolonged period.^1^ They can pose a huge burden on the health care system including dialysis facilities. The implications of the health consequences of central obesity can be lucidly and effectively conveyed to the public through school health programs.

## Treatment of Metabolic Syndrome

Gradual reduction of body weight to achieve BMI as close as possible to the normal range is the cornerstone of therapy. Lifestyle modifications are vital. These include reducing the energy intake, low intake of saturated fats, trans fats, cholesterol, and simple sugars along with higher intake of monosaturated fats, fresh fruits, vegetables, and whole grains. Complete cessation of smoking and alcohol intake in moderation is mandatory. Moderate-intensity exercise daily for 30 min is beneficial.

Blood pressure (BP) should be controlled optimally. angiotensin converting enzyme (ACE) inhibitors and angiotensin receptor blocker (ARBs) are better first-line therapy for metabolic syndrome, especially when diabetes is present.^2^ Besides telmisartan has the added advantage of having a partial peroxisome proliferator-activated receptor (PPAR) γ agonist activity.^19^ Metformin and thiazolidenidiones (PPARγ agonist) improve insulin sensitivity and reduce the risk of type 2 diabetes in patients with impaired fasting glucose (IFG) or impaired glucose tolerance (IGT).^2^ However, a recent report of increased myocardial infarction with rosiglitazone is disconcerting.^20^ Fibrates which are PPARα ligands^21^ are recommended for hyperglyceridemia, while statins may be used in hypercholesterolemia.

## Newer Therapies

Recently Rimonabant, a cannabinoid receptor type 1 antagonist, has shown promising results in type 2 diabetics as well as nondiabetics with obesity.^22^–^24^ It significantly reduces weight and waist circumference. Metabolic changes including reduced prevalence of metabolic syndrome, reduced fasting glucose, improved lipid profile, and elevated adiponectin have also been demonstrated. However, long-term studies are needed before definite recommendations can be made.

Soy protein has been found to have therapeutic significance in reducing severity of diabetes, metabolic syndrome, and renal dysfunction in obese Zucker rats.^25^ Detailed human studies are awaited.

PPARδ may be a new therapeutic target for metabolic syndrome.^21^ PPARδ ligand GW610742 induces energy dissipation in skeletal muscles and adipose tissue; and reduces hepatic glucose output by increasing glycolysis. Thus it seems to hit at the very roots of the pathogenetic mechanism of metabolic syndrome. Beneficial effect on metabolic syndrome has been confirmed in animal models.

## Surgical Therapy

Bariatric surgery is being increasingly done for morbidly obese individuals, who fail medical therapy. It has been shown to be effective in treating metabolic syndrome. There is a decrease in insulin resistance as a result of weight loss and increased secretion of gut hormones like Glucagon-like-peptide-1.^26^
